# Visual Features Underlying Perceived Brightness as Revealed by Classification Images

**DOI:** 10.1371/journal.pone.0007432

**Published:** 2009-10-13

**Authors:** Ilmari Kurki, Tarja Peromaa, Aapo Hyvärinen, Jussi Saarinen

**Affiliations:** 1 Department of Psychology, University of Helsinki, Helsinki, Finland; 2 Department of Computer Science and HIIT, University of Helsinki, Helsinki, Finland; 3 Department of Mathematics and Statistics, University of Helsinki, Helsinki, Finland; New York University, United States of America

## Abstract

Along with physical luminance, the perceived brightness is known to depend on the spatial structure of the stimulus. Often it is assumed that neural computation of the brightness is based on the analysis of luminance borders of the stimulus. However, this has not been tested directly. We introduce a new variant of the psychophysical reverse-correlation or classification image method to estimate and localize the physical features of the stimuli which correlate with the perceived brightness, using a brightness-matching task. We derive classification images for the illusory Craik-O'Brien-Cornsweet stimulus and a “real” uniform step stimulus. For both stimuli, classification images reveal a positive peak at the stimulus border, along with a negative peak at the background, but are flat at the center of the stimulus, suggesting that brightness is determined solely by the border information. Features in the perceptually completed area in the Craik-O'Brien-Cornsweet do not contribute to its brightness, nor could we see low-frequency boosting, which has been offered as an explanation for the illusion. Tuning of the classification image profiles changes remarkably little with stimulus size. This supports the idea that only certain spatial scales are used for computing the brightness of a surface.

## Introduction

**Figure 1:**
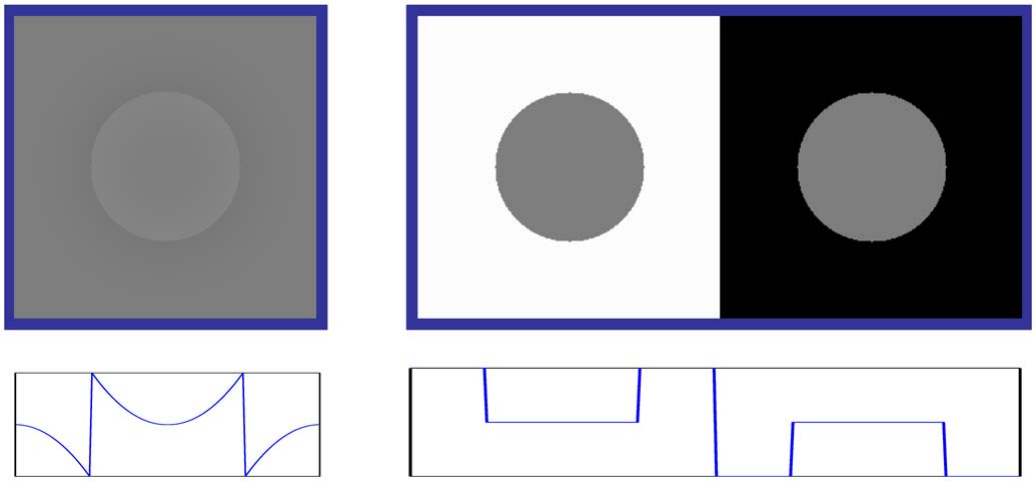
Classical brightness illusions demonstrate that spatial properties of the scene, such as luminance gradients and ratios between adjacent areas play a dramatic role in brightness perception. Luminance profiles are shown under the figures. Leftt: Craik-O'Brien-Cornsweet illusion. The luminance gradient at the border elicits an illusion of a uniform bright surface even when there is no physical luminance difference at the center of the patch. Right: Simultaneous brightness contrast illusion Central patches are of the same gray value, but the right patch with black background appears brighter than the left with white background.

It is well known that along with physical luminance, the perceived brightness depends on the spatial structure of the stimulus. Luminance borders (discontinuities) are known to modulate brightness (for a review, see for example [1], [2]). A classical demonstration of how the luminance profile of the border affects the perceived brightness is the Craik-O'Brien-Cornsweet illusion, where a slowly changing luminance gradient is able to produce a percept of a uniform bright surface even when the luminance in the center of the stimulus is actually exactly the same as outside of the stimulus. Similarly, contrast with the background luminance can increase the perceived brightness of the patch, as in the simultaneous contrast illusion (figure 1).

Since the border information is known to dramatically alter the perceived brightness, many models assume that the borders of the stimulus are critical for the surface representation. The cells in the primary visual cortex are known to respond best to local luminance discontinuities (borders and outlines), but give little or no response to the uniform luminance [3]. The first stage in many brightness models consists of filtering the input stimulus by local, spatial frequency selective neural filters. In the following stages the local filter responses are integrated to a surface representation. For example, in the model of Morrone and Burr [4], the filter responses are analyzed into a symbolic representation of “bar” and “edge” descriptors. Another scheme is the neural “filling-in” models, where the properties of surface are computed in a neural network. Neurons at the border are assumed to send a “filling-in” signal to the cells in the center of the surface [5], [6].

Despite decades of theoretical development of brightness models, it has been challenging to empirically evaluate assumptions underlying the models. Typically, brightness models are evaluated on the basis of their ability to explain various visual illusions. However, this indirect approach is not very informative about the actual stimulus information processing that takes place in the visual system, e.g. what parts and features of the stimulus are critical in the brightness computation. The approach here is to empirically measure the information in the stimulus that correlates with perceived brightness. We use a new psychophysical reverse correlation method known as classification images [7]–[9] that allows both localization and characterization of the stimulus information that correlates with perceived brightness. Aside from a few studies [4], [10] that have used noise-masking techniques to estimate the spatial frequency tuning of the critical stimulus information in brightness, there is little direct evidence on what information is used when computing the brightness of an extended surface.

**Figure 2:**
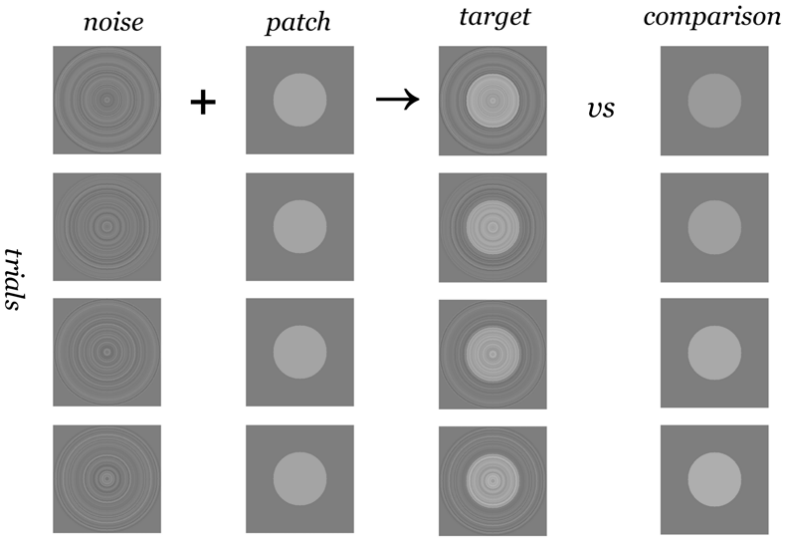
Stimuli and procedure. A forced-choice luminance matching procedure was used. Two stimulus intervals were shown in random order to the subject. The target stimulus consisted of a constant luminance patch masked by low-contrast ‘ring’ white noise. The luminance of the unmasked comparison patch was varied (4 levels). The subject's task was to indicate the interval in which the patch appeared brighter.

The classification image method is based on masking the target stimulus (a uniform luminance “step” patch, or a Craik-O'Brien-Cornsweet stimulus, see fig. 2) with a random white noise stimulus that is created anew in every trial. This causes slight fluctuations in the perceived brightness of the patch, depending on how the random visual features within the white noise match the (unknown) filters that are used in the task. In the classification image analysis, the correlation between the intensity of each point (stimulus ring) of the noise and the subject's perceptual decision (here, how bright the patch appears) is computed, resulting in a correlation map or “behavioral receptive field” that reveals the parts of the target stimulus that the subject uses to carry out the task. Classification images allow investigation of perceptual processing directly in the spatial domain, as opposed to e.g. the spatial frequency -masking method that gives information only about the spatial frequency tuning of the processing.

The area of the rings grows as a function of the radius. The classification image reveals how much total weight the subjects give to the stimulus information in different rings. This is dependent on sensitivity of the perceptual mechanism multiplied by the area, i.e. its extent on the ring. However, a more common way to characterize the sensitivity of perceptual mechanism is to compute the sensitivity per unit area, giving the cross-section of the underlying “behavioral receptive field”. Therefore we analyzed also the weights per unit area by normalizing the classification images by the area of each ring. This also ensures that our results do not reflect simply sensitivity to the area of the signal rings.

**Figure 3:**
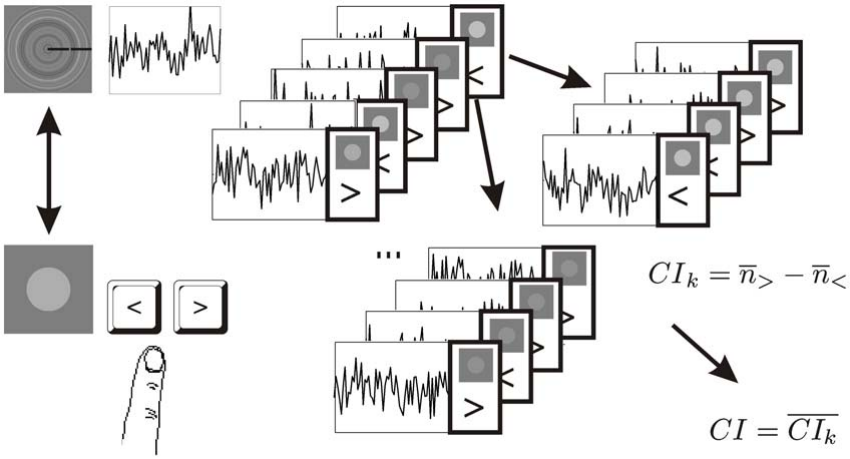
Data analysis. Classification images were computed in two steps. First, sub-classification images for each comparison luminance level were computed by taking an average (across the trials) of the noise masks with “comparison brighter than the target”. This was subtracted from the average of noise masks with “comparison not brighter than the target”, resulting in four sub-classification images for each comparison luminance. The classification image was computed by taking the average of the sub-classification images.

Classification images were measured using a two-interval method of constant stimuli. A masked test patch of constant luminance and a comparison stimulus of varying luminance (4 levels) were presented in random order. Subjects chose the interval in which the patch appeared brighter (see figure 3). Contrary to some previous studies aiming at characterizing the brightness processing [11], [12], we used a subjective matching task and not a forced-choice task — this ensures that the classification images estimate the information that correlates with perceived brightness and not e.g. with better visibility or detectability of the target stimulus.

In the first experiment, we studied the visual features that the subject uses when judging brightness. In particular, we compared the processing of Craik-O'Brien-Cornsweet and step stimuli, using a 1.33 deg target radius. The similar appearance of an illusory Craik-O'Brien-Cornsweet -surface and a real luminance (“step”) surface has been suggested to imply that also real extended surfaces are in a sense illusory — that only the borders are processed and the representation of the middle of the surface is based on interpolation or “filling-in” rather than direct sensory input. This hypothesis, however, has not been previously tested directly. A low-contrast Craik-O'Brien-Cornsweet stimulus was used to ensure that stimulus is able to elicit an illusion of a surface as it is known to diminish on high contrast. The strength of the illusion was quantified by estimating the perceived luminance (see [13]) of the Craik-O'Brien-Cornsweet surface. The proportion of “brighter than” judgments was computed for each comparison stimulus luminance level and a psychometric function was fitted to the data. The value corresponding to the 50% point in the function was used as a point of subjective equality.

In the second experiment, we tested how the visual information utilized in brightness perception depends on the size of the target (radius 0.33—1.33 deg). Sharp-edged stimuli are broad-band in terms of spatial frequency content (here, >6 oct) whereas filters in early visual areas are known to have quite narrow spatial frequency tuning (ca. 1 octave) [14]. What spatial frequencies of the stimulus are used for computing the brightness? By decreasing the stimulus size, the spatial frequency band of the border is shifted towards higher spatial frequencies, thus changing the spatial frequency tuning of “available” information. It has been shown that in tasks such as letter identification [15], [16] and face recognition [17], [18], just a very limited scale of spatial frequencies of the target is utilized. Studies in brightness perception suggest that the spatial scale critical for brightness is either constant [19] or changes slightly with the size of the stimulus[20]. Also the particular spatial scales suggested vary — in some studies, the critical scales occur at low spatial frequency range (≈1 cpd) [10], [19] and in others, at medium spatial frequency range (1.5—5 cpd) [20]. By using a particularly simple stimulus (as compared to complex stimuli in studies [10] and [19]) and a task directly measuring brightness perception (as compared to an indirect task in [20]), we hope to clarify the issue.

## Results

**Figure 4:**
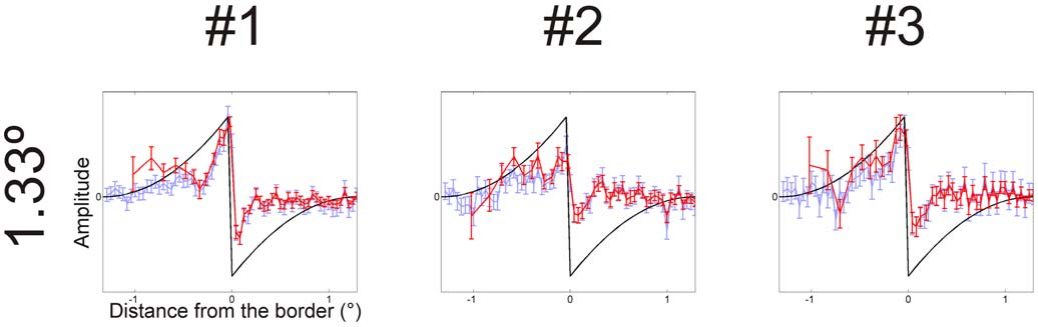
Classification images: Craik-O'Brien-Cornsweet stimulus for three subjects. The black line shows the target profile. The blue curve is “raw” classification image and the red curve the spatially normalized classification images divided by the area of the stimulus rings. The classification image profile reveals a positive peak at the location of the border. The amplitude is nonzero just at the border, while the illusory area is almost flat. This implies that subjects rely on the stimulus information at the border when assessing the brightness of the surface. The Error bars: 1 standard error of the mean.

In the first experiment, a Craik-O'Brien-Cornsweet profile (radius 1.33 deg) was used. Results (“raw” and “normalized” classification images) are shown in figure 4. Classification images peak inside the border of the patch and have negative peaks in the background, next to the border. The tuning of the underlying mechanism can be estimated from normalized classification image. The positive lobe is narrower than the stimulus profile; the amplitude drops close to zero in the “illusory” area farther from the border.

The mean perceived (point of subjective equality) brightness of the Craik-O'Brien-Cornsweet stimulus was 53.9 cd/m^2^. This corresponds with 77% of the peak luminance of the Craik-O'Brien-Cornsweet (55 cd/m^2^) thus suggesting that subjects perceived a vivid illusion of a bright surface (see [13]).

**Figure 5:**
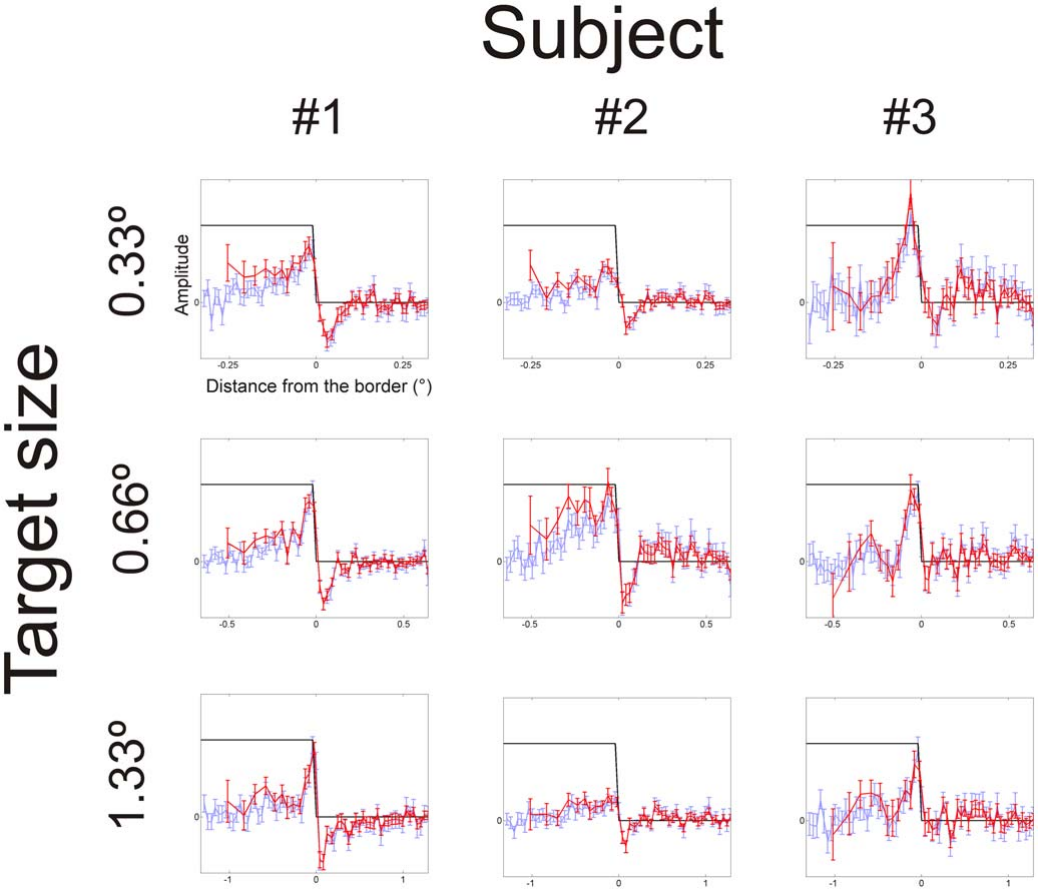
Classification images: step edge. The black line shows the target profile. The blue curve is the “raw” classification image and the red curve the spatially normalized classification image. Classification image profiles peak inside the stimulus, at the location of the border. In most cases, there is also a negative peak at the background next to the border. The relative extent of the classification image profile compared to the target size is dependent on the size: with small 0.33 degree stimulus it encompasses the entire stimulus, but with large 1.33 degree stimulus, it covers just the border.

In the second experiment, we derived classification images for a uniform surface stimulus with “step” – profile (figure 2). The radius of the target was varied: 0.33, 0.66 or 1.33 deg. Classification images for three subjects are presented in figure 5. Classification images reveals positive peaks inside the border of the patch and less prominent negative peaks in the background immediately next to the border. The spatial-frequency tuning of the underlying mechanism (as estimated from normalized classification images) is band-pass. With the smallest 0.33 target, the lack of highest frequencies is clearly visible, as the amplitude drop in the border is gradual rather than sharp. The lack of the lowest spatial frequencies can be seen clearly from 0.66 and 1.33 deg target figures, as the amplitude of the classification image drops to zero farther from the border.

To characterize further the tuning, odd-symmetric exponential functions were fitted to the excitatory lobes of the normalized classification image profiles. Estimated from the Fourier-transformation, the tuning peaks at 3.0, 3.0 and 2.6 cpd (subject #1) and 6.1, 3.8 and 2.3 cpd (#2) when the stimulus radius was varied from 0.33 to 1.33 deg. The low-frequency cut-offs were 0.79, 0.84 and 0.84 cpd (#1) 1.5, 1.1 and 0.64 cpd (#2). Exponential functions did not fit well to classification image profiles of subject #3, this method could not be used to characterize the tuning.

**Figure 6:**
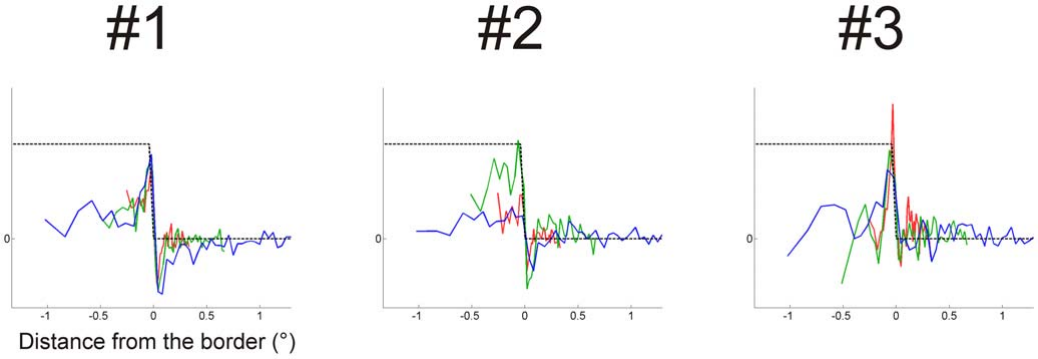
Normalized classification images for step edge: comparison of different target sizes. Blue curve: 1.33 deg target, green curve: 0.66 deg, red curve: 0.33 deg target. Tuning of the classification image profile is almost invariant to the target size.

The tuning of the normalized classification images is best seen when plotted to the same coordinates (figure 6). Larger stimuli have slightly wider profiles, but the profiles largely overlap, implying that four-fold increase in the stimulus size has a remarkably small effect to the tuning.

## Discussion

In summary, we found that (1) profiles of the classification images for perceived brightness peak at the border of the patch. The peak rapidly attenuates as a function of distance from the border. (2) Classification image profiles for Craik-O'Brien-Cornsweet or step stimuli have no consistent features in the center of the surface (perceptually completed area) and (3) the classification image profiles scale just slightly with the size of the stimulus.

The results show that brightness is determined solely by the border information; the area further from the border does not seem to contribute to it, neither in the case of perceptually completed surface of the Craik-O'Brien-Cornsweet nor the luminance signal in the step edge (second experiment). This gives direct support for the idea that surfaces are interpolated or ‘filled-in’ from the border information. Even when the center of the surface contains a luminance signal, as in the case of the step edge, it is not used for brightness computation. In fact, the classification images are very similar for both real (step edge) and illusory (Craik-O'Brien-Cornsweet) stimuli.

In terms of filter-based brightness models, the result implies that brightness is computed from responses of odd-symmetric “edge” detectors. Neural filling-in models also predict that border information should play a major role in brightness. However, it is often [5], [6] assumed that along with the border information, a neural channel sensitive to the absolute luminance level exists. We could not find support for this assumption: the (absolute) luminance response outside the border is very weak.

Recently, Dakin and Bex have offered a channel re-weighting/low-frequency boosting model to explain the Craik-O'Brien-Cornsweet illusion [11]. The spatial frequency structure of natural images is known to have a characteristic 1/f structure – the average amplitude of the spatial frequency components drops as a function of the spatial frequency (*f*). The model proposes that the visual system re-weights the spatial frequency channels so that their output is matched towards the expected 1/f structure. In the cases where low spatial frequency information is weak (as in the case of Craik-O'Brien-Cornsweet) this implies boosting of low frequencies, explaining the surface-like appearance. Dakin and Bex presented evidence for the boosting by measuring classification image for contrast polarity discrimination for Craik-O'Brien-Cornsweet annulus (width 0.27 deg). The edge in the classification image profile was wider than in the stimulus profile and spread out to the ‘filled-in’ area that contained no luminance signal. This was interpreted as boosting of low spatial frequencies in the stimulus [11]. Our results are very different; the classification image profile of the illusory stimulus was not wider than target. This is clearly against the idea that perception of the Craik-O'Brien-Cornsweet surface is mediated by boosting of low-frequency stimulus information.

If the low-frequency boosting is not the explanation for the Craik-O'Brien-Cornsweet illusion, why it was observed in [11]? The very small target used by Dakin and Bex is arguably not well suited for investigating surface perception, since small stimuli can be perceived by means of local mechanisms without necessity of integrating the responses across the space. Apparent boosting of low-spatial frequency content in this very high-frequency stimulus can be totally unrelated to surface perception, and could result in e.g. from low-pass optical filtering due to the optical qualities of the eye [21]. We also point out that a spreading of the classification image profile does not necessarily imply low-frequency boosting. In the standard classification image analysis used, it is assumed that the observer has complete knowledge of the location and profile of the target stimulus. However, with a tiny stimulus at the discrimination threshold this may not be a realistic assumption. Tjan and Nandy [22] showed that the spatial uncertainty can induce low-contrast “haze” to classification images, which can be erroneously interpreted as low-frequency features.

A number of studies have recently investigated the spatial frequency tuning of brightness perception by a psychophysical masking/filtering paradigm, where the spatial frequency content of the stimulus is manipulated either by filtering the stimulus [19] or by masking it with filtered visual noise [10], [20]. Both methods have suggested spatial frequency selective processing with only certain frequencies contributing to the brightness. Perna and Morrone [19] reported that removing the frequencies at around 1 cpd decreased dramatically the perceived brightness as measured by a brightness matching task. Scaling was investigated by high-pass filtering the target stimuli of variable size. The critical cut-off frequency was independent of the stimulus size [19]. Salmela and Laurinen [20] used a band-pass masking paradigm and reported that the tuning function in the contrast polarity discrimination task was 4, 2.2 and 1.7 cpd for stimulus sizes of 0.2, 0.8 and 3.3°. With very large stimuli, spatial frequency tuning of the masking was found to be almost independent of the size of the stimulus. Our results suggest also that the tuning of brightness mechanisms is almost independent on the stimulus size and has band-pass characteristics. The tuning of the classification image profiles are consistent with the idea that low- and medium spatial frequencies are used for brightness perception [10], [19], [20] and the low-frequency cut-off might be around 1cpd [19].

Lastly, it is interesting to compare the results of the second experiment to the results by Shimozaki, Eckstein and Abbey [12], who studied contrast discrimination of similar step stimuli (radius 0.68 deg) and radial noise as here. Classification image profiles peaked at the border of the stimulus and background, with a reverse-signed “inhibitory” lobe in the surround. The amplitude of the (not normalized) profile was inversely proportional to the distance from the border, spanning ca. 0.25 deg and dropping close to zero near the center of the circular surface, thus similar to the matching-classification images found here. This suggests that both perceived contrast and brightness of simple surface stimulus are mediated by similar mechanisms responding to the borders of the stimulus (see also [23], [24]).

As a conclusion, we used the psychophysical classification image method with brightness matching task to measure the stimulus features of the uniform step or Craik-O'Brien-Cornsweet stimulus that correlate with perceived brightness. Classification images peak at the border of the surface and the amplitude of the classification image attenuates rapidly towards the center of the stimulus. This suggests that the brightness of an extended surface is determined by the border information, supporting the idea that perception of surfaces is based on ‘filling-in’ the surface information from the borders. Classification images had clearly band-pass rather than low-pass tuning, problematic for luminance channel and low-frequency boosting assumptions in some models. Changing the stimulus size reveals that the the tuning of the mechanisms underlying the perceived brightness is largely independent on the size of the stimulus. Using classification images with a task where subjects have to explicitly assess the attribute of interest can be used to obtain direct and rich new information about processing of more complex attributes of the stimulus, such as brightness, (see also:[25], [26]).

## Materials and Methods

### Apparatus and Stimuli

Psychophysical experiments were conducted in a dimly lit room. Stimuli were generated using ViSaGe stimulus generator (Cambridge Research Systems, Rochester, UK) and displayed on a calibrated Mitsubishi Diamond Pro 2070SB monitor (display size 800×600 pixels, 39×29.3 cm, at 100 Hz refresh rate). The mean luminance of the display was 50 cd/m^2^. Stimuli were generated with Matlab 7 (MathWorks Inc, Natcik, MA).

Comparison stimuli (Fig. 2) consisted of uniform circular patches (radius 32 pixels, 0.33, 0.67 or 1.33 deg) whose luminance was varied (4 levels, chosen so that the lowest luminance resulted in ca. 10% brighter-than judgments and the highest ca. 90%. During the experiments, slight adjustments of luminance levels were occasionally done in order to maintain these conditions. Comparison stimulus luminance varied between c.a. 52 and 56 cd/m^2^ for the Craik-O'Brien-Cornsweet stimulus and between and between c.a. 61 and 72 cd/m^2^ for the step stimulus.)

In the first experiment, the test patch was a circular Craik-O'Brien-Cornsweet stimulus, generated using a parabola, width at half height 0.37 deg. The Michelson contrast of the patch was 10% (peak luminance 55 cd/m^2^, luminance at the center 50 cd/m^2^.) Low contrast was used as it is known that the Craik-O'Brien-Cornsweet illusion persists only at low contrast levels. The test patch was masked with annular “ring” noise (Fig. 2) consisting of 64 concentric rings, total radius 2.67 deg. The luminance of the rings was selected independently from a Gaussian distribution (s.d. 2 cd/m^2^ or 4% rms-contrast). Comparison (step) patch had the same radius as the test.

In the second experiment, the test patch was a circular patch whose radius was varied (0.33, 0.67 or 1.33 deg), masked with annular “ring” noise (s.d. 3 cd/m^2^). Comparison patch had the same radius as the test. The size of the stimuli was varied by adjusting the size of the pixels (1×1 or 2×2) and viewing distance. The peak luminance of the test patch was 65 cd/m^2^. To hasten the data collection, the peak luminance (and the noise standard deviation) for the step stimulus was higher than for the Craik-O'Brien-Cornsweet. The values of target and noise energies were selected on the basis of the patch being clearly above detection threshold, but the superimposed noise masks still had a noticeable effect on the perceived brightness of the patch.

### Subjects

Subjects were volunteers and received no compensation for taking part to the study. All had normal vision. All subjects had previous experience of psychophysical experiments. #1 is one of the authors, while #2 and #3 were not aware of the purpose of this study. Before the main experiments, all subjects were trained in the brightness matching task without noise masks in the test stimulus. Participants gave written informed consent.

### Procedure

The method of constant stimuli (MOCS) with a two-interval brightness matching task was used. Subjects were instructed to choose the interval in which the patch appeared brighter. Since the masked standard patch appears slightly non-homogeneous, it was emphasized to judge the brightness of the patch “as a whole” rather than the peak brightness of the patch and the noise.

The presentation order of the standard and comparison stimulus was randomized. The trial started with a fixation crosshair displayed for 200 ms, followed by a blank screen for another 200 ms. Then the first stimulus was shown for 200 ms, followed by an inter-stimulus-interval (400 ms), after which the second stimulus was shown for 200 ms. After that, the subject chose the interval in which the patch appeared brighter by pressing a key. The next trial followed immediately.

Experiments were done in blocks of 100 trials of the same target radius and profile. The data is based on at least 2,000 trials for each condition and subject (typically: 4,000). The experiments took ca. 6 hours to complete for each subject, conducted during several days.

The procedure was approved by the Committee for Research Ethics of the Faculty of Behavioral sciences in University of Helsinki.

### Classification image analysis

The standard classification image analysis [7] was used, except for first computing separate sub-classification images for each comparison stimulus level. The noise masks superimposed onto the test stimulus, comparison stimulus luminance and subject's response (test/comparison brighter) was recorded for each trial. The sub-classification images were computed for each comparison stimulus luminance level *k* by taking the point-wise average of the noise masks *n* with “brighter than” - judgments *“>”* in given comparison stimulus luminance and subtracting from it the average of the noise masks with “not brighter than” - judgments *“<”*.
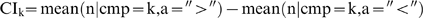
(1)


Then, the classification image was computed by taking the mean over sub-classification images.
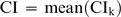
(2)


Stimulus rings close to the center and close to the border have different spatial areas. To ensure that the classification image profiles are not distorted by this difference of spatial areas, we computed normalized classification images by dividing each point in the classification image by the area of the stimulus ring (number of the pixels). On the other hand, this normalization makes the classification image profiles very noisy in the most central points (rings) that had area of only a few pixels. To reduce the noise, neighboring points in the normalized classification image were averaged. Number of the neighboring points in each bin of the spatial average was fixed to have an equal stimulus area of at least 256 pixels^2^. This causes coarser averaging in the center of the stimulus, where the estimation noise was worst. The validity of the estimation method was confirmed by computer simulations. Details of the analysis and simulations can be obtained from IK upon request.

Confidence intervals were then computed using Bootstrap methods [27]. The original data was partitioned to sets by the comparison stimulus luminance and the answer. Each set was then randomly sampled (with replacement) to generate a bootstrap replica. Classification images were then computed using formulas (1) and (2) using 2048 replicas. The standard deviation of the Bootstrap replicas was used as an estimate of the standard error of the classification images.

### Estimation of the perceived brightness

For the Craik-O'Brien-Cornsweet experiment, the perceived brightness of the target was quantified by finding the point of subjective equality of the comparison stimulus luminance that corresponds to the perceived luminance of the Craik-O'Brien-Cornsweet stimulus. The proportion of “brighter than” judgements was calculated for each comparison luminance level. A psychometric function was then fitted to this data. The point of subjective equality, an estimate of the perceived brightness of the Craik-O'Brien-Cornsweet corresponds to the 50% point of the psychometric function. A fitting procedure was done separately for blocks of 100 trials and the final estimate is the mean of these.
